# Structural Statistical Quantifiers and Thermal Features of Quantum Systems

**DOI:** 10.3390/e23010019

**Published:** 2020-12-25

**Authors:** Flavia Pennini, Angelo Plastino, Angel Ricardo Plastino, Alberto Hernando

**Affiliations:** 1 Departamento de Física, Universidad Católica del Norte, Av. Angamos 0610, Antofagasta 1270709, Chile; fpennini@ucn.cl; 2 Departamento de Física, Facultad de Ciencias Exactas y Naturales, Universidad Nacional de La Pampa, CONICET, Av. Peru 151, Santa Rosa, La Pampa 6300, Argentina; 3 Facultad de Ciencias Exactas, CONICET, Universidad Nacional de La Plata, C.C. 67, La Plata 1900, Argentina; angeloplastino@gmail.com; 4 Kido Dynamics SA, Avenue de Sevelin 46, 1004 Lausanne, Switzerland; 5 CeBio y Departamento de Ciencias Básicas, Universidad Nacional del Noroeste de la Prov. de Buenos Aires, UNNOBA, CONICET, Roque Saenz-Peña 456, Junin 6000, Argentina; arplastino@unnoba.edu.ar

**Keywords:** thermal uncertainties, disequilibrium, semi-classical distributions

## Abstract

This paper deals primarily with relatively novel thermal quantifiers called disequilibrium and statistical complexity, whose role is growing in different disciplines of physics and other sciences. These quantifiers are called L. Ruiz, Mancini, and Calvet (LMC) quantifiers, following the initials of the three authors who advanced them. We wish to establish information-theoretical bridges between LMC structural quantifiers and (1) Thermal Heisenberg uncertainties ΔxΔp (at temperature *T*); (2) A nuclear physics fermion model. Having achieved such purposes, we determine to what an extent our bridges can be extended to both the semi-classical and classical realms. In addition, we find a strict bound relating a special LMC structural quantifier to quantum uncertainties.

## 1. Introduction

The motivation for studying L. Ruiz, Mancini, and Calvet (LMC) structural quantifiers (LMCSQs) in quantum mechanics is that these measures have been recently shown to describe important aspects of quantum systems at zero temperature [1,2,3]. Consequently, it would be interesting to analyze how LMCSQs behave at finite temperatures.

### 1.1. LMC Structural Quantifiers

LMCSQs have become important tools in several scientific disciplines [1,2,3,4,5,6,7,8,9,10,11,12,13,14,15].

In most systems, a certain level of randomness (usually quantified by an entropy *S*) coexists with some number of correlation structures. This fact can be viewed as an intermediate stage between two opposite extreme situations: (A) perfect order or (B) maximal randomness (no correlations exist). This intermediate stage has been successfully quantified in the last 20 years by a quantity that came to be called the statistical complexity *C*—advanced in Ref. [4]—which can be properly regarded as a structure–content quantifier [1,2,3,4,5,6,7,8,9,10,11,12,13,14,15]. In Ref. [4], the authors established a kind of “distance” in probability space (PS) that they referred to as the disequilibrium *D*. What does it measure? If *f* is the probability density that describes the system at hand and $f_{u}$ is the uniform probability density, then *D* tells us how far the two distributions, f(x) and $f_{u}$, differ from each other [5]. In density matrix parlance, *D* measures the distance between the extant density matrix and the maximally mixed one and is associated with order, which grows with the value of *D*.

(1) $$D=\int dx{\left(f\left(x\right)-f_{u}\right)}^{2}.$$

In addition, *D* provides a notion of hierarchy that makes it non-null if there are privileged states among the accessible ones. *D* would then be maximal for (A) and vanish for (B) above. In the entropy case, things are exactly reversed. *S* is minimal for (A), while it reaches a maximum for (B). Reasoning in this way, L. Ruiz, Mancini, and Calvet (LMC) [4,5,6] formulated what constitutes today the standard way of casting a statistical complexity measure or structure–content quantifier *C*, which is written as a product between the entropy *S* and *D*:

(2) C=DS;S=−∫dxf(x)ln(x),

a functional of the density distributions (DDs) [4]. This proposal received great attention (see Refs. [1,2,3,4,5,6,7,8,9,10,11,12,13,14,15] as a small sample). It was used in different scenarios for the canonical, microcanonical, and grand canonical ensembles. As already mentioned, in the present context, we refer to *C* as a structure–content quantifier because one of the systems to be discussed is the harmonic oscillator, which by no means can be regarded as “complex”. In fact, *C* has been of utility in the realm of non-complex systems (see Refs. [2,3] and the references therein).

The above thermal quantifiers will be applied below to a nuclear physics model that has attracted attention [16,17,18,19,20,21,22,23,24,25].

### 1.2. Thermal Uncertainty Relations (TURs)

The motivation for this TUR endeavor is based on a recent discussion by Nagata [26], who analyzed finite-temperature uncertainties and their relation with the LMC structural quantifiers *C* (statistical complexity) and *D* (disequilibrium).

Thermal uncertainty relations (TURs) were the subject of great efforts and exceedingly interesting work (one can look, for instance, at [26,27,28,29,30,31,32,33]. A recommendable review was provided by Uffink and van Lith [34]. These thermal uncertainties [35] will be the focus of the present work, particularly in connection with the disequilibrium notion, which will be explained below. Our motivation arises from consideration of the TURs. Within this framework, we wish to encompass the behavior of the LMC structural quantifiers [1,2,3,4,5,6,7,8,9,10,11,12,13,14,15].

## 2. The Thermal Quantum Case

With regards to quantum mixed one-dimensional states, after consulting and relating references [5,26,36,37], to see if one can cast the pertinent density matrix $\hat{\rho }$ and the associated disequilibrium *D* in a simple fashion, one starts with

(3) $$\hat{\rho }=\left(1-e^{-\beta \hbar \omega }\right)e^{-\beta \hat{n}},$$

where $\beta =1/k_{B}T$, $k_{B}$ the Boltzmann constant, and $\hat{n}$ is the number operator [36]. In this paper, we set the Boltzmann constant equal to unity ($k_{B}=1$). Then, one can express the quantum disequilibrium in the fashion [5,36,37]:

(4) $$D=\mathrm{Tr}{\hat{\rho }}^{2}.$$

Note that here *D* is exactly equal to the purity (or degree of mixedness) P(⊂) of $\hat{\rho }$, so that 0≤D≤1. With some simple manipulations, one can also ascertain that [5,26,36,37]:

(5) D=tanh(βħω/2).

Further, the quantal harmonic oscillator (HO) expression for the entropy *S* is [36]:

(6) $$S=\frac{\beta \hbar \omega }{e^{\beta \hbar \omega }-1}-\ln\left(1-e^{-\beta \hbar \omega }\right),$$

so that, with *S* and *D* at hand, the quantum structural quantifier C=DS becomes

(7) $$C=\tanh\left(\beta \hbar \omega /2\right)\left(\frac{\beta \hbar \omega }{e^{\beta \hbar \omega }-1}-\ln\left(1-e^{-\beta \hbar \omega }\right)\right),$$

which vanishes both at T=0 and at T=∞, as one should expect. In addition, we believe it convenient to add here the useful well-known HO expressions for the Helmholtz free energy *F*, the mean value of energy *U*, and the specific heat $C_{V}$ [36]:

(8) $$F=\frac{\hbar \omega }{2}+T\ln\left(1-e^{-\beta \hbar \omega }\right)$$

(9) $$U=\langle \hat{H}\rangle =\hbar \omega \left(\langle \hat{n}\rangle +\frac{1}{2}\right)\equiv \frac{\hbar \omega }{2\tanh(\beta \hbar \omega /2)}$$

(10) $$C_{V}={\left(\frac{\hbar \omega \beta }{e^{\beta \hbar \omega }-1}\right)}^{2}e^{\beta \hbar \omega }-.$$

Finally, the the mean particle number $\langle \hat{n}\rangle$ is easily seen to be

(11) $$\langle \hat{n}\rangle =\frac{1}{e^{\beta \hbar \omega }-1}.$$

We now have enough material to derive our desired equality below.

## 3. A Strict Bound Relating *D* to Quantum Uncertainties

Our present results arise at this stage. We remind the reader that *D*, which is equal to the ratio C/S, can also be regarded as the ratio between a structural and a random quantifier. The thermal Heisenberg uncertainty relation is of the form [35,37]:

(12) $$\Delta x\;\Delta p=\frac{\hbar }{2}\;\coth\left(\beta \hbar \omega /2\right),$$

so that it can be cast in the fashion:

(13) $$D=\frac{\hbar /2}{\Delta x\;\Delta p}=U_{r},$$

where Δx and Δp are the quantum variances for the canonically conjugated observables *x* and *p* [37], and thus, *D* equals the ratio $U_{r}=\left(\hbar \omega /2\right)/U$ between the minimum possible uncertainty value (attributable to coherent states) and the actual uncertainty value of the mixed state under consideration. This ratio, in turn, is also the purity $P(\hat{\subset })$. We are then immediately led to our first significant result (we repeat that *D*, which is equal to the ratio C/S, can also be regarded as the ratio between a structural and a random quantifier):

(14) $$D\;\;\Delta x\;\Delta p=\frac{\hbar }{2}.$$

Surprisingly enough, there exist semi-classical and even classical counterparts of the above equality, as we will show below.

Equation (14) can also be cast in purity terms, in the fashion:

(15) $$P(\subset )\;\;\Delta x\;\Delta p=\frac{\hbar }{2},$$

which constitutes, let us insist, a strict quantum equality (for the HO), which we believe to have newly established here. This relation also tells us that the ratio of “structure/randomness” times thermal uncertainty equals ħ/2.

**Figure 1:**
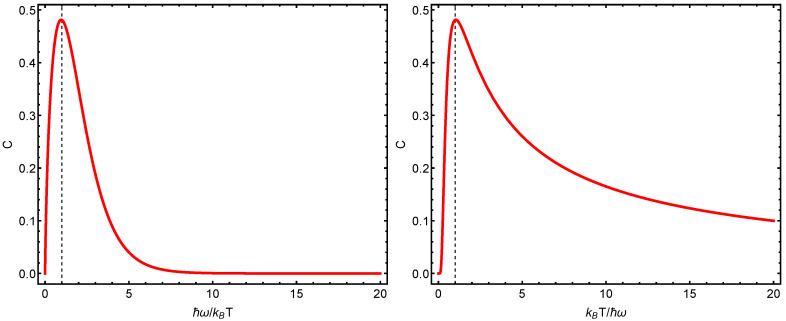
(**Left panel**): statistical complexity *C* versus $\hbar \omega /k_{B}T$. The maximum *C* value is detected whenever the thermal energy equals the vibrational one, which happens at $\hbar \omega /k_{B}T=1$, as indicated by the vertical line. (**Right panel**): *C* versus $k_{B}T/\hbar \omega$. In addition, the maximum is located in $\hbar \omega /k_{B}T=1$.

**Figure 2:**
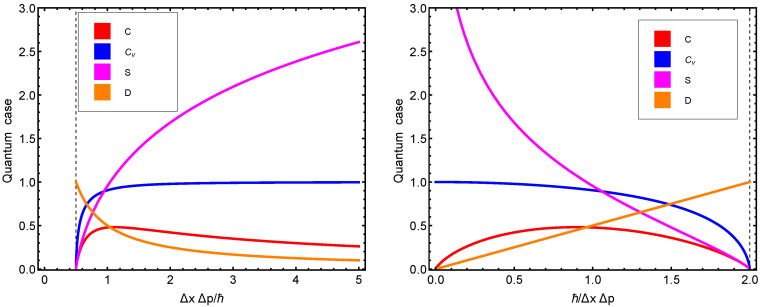
(**Left panel**): thermal quantum quantifiers (TQFs) versus ΔxΔp/ħ. Towards the right, we reach the classical limit. We can appreciate how the thermal quantifiers behave along such a route. (**Right panel**): TQFs versus ħ/ΔxΔp expressed in $k_{B}$-units. Towards the left, we reach the classical limit. These plots should be compared to the corresponding ones displayed in Ref. [26].

We now depict some statistical quantifiers versus either Heisenberg’s uncertainty $q_{1}=\Delta x\Delta p/\hbar$ or $q_{2}=\hbar \omega /k_{B}T$. We begin with von Neumann’s entropy in Figure 1, which exhibits maxima at $q_{1}=1=q_{2}$. Figure 2 displays several thermal quantifiers versus uncertainty. These curves will be compared below with their semi-classical Husimi counterparts.

We highlight here this fact regarding the right panel: The quantum structural quantifier does not attain its maximum at minimal uncertainty ħ/2, but at twice this value. This value is the minimum one that can be reached for semi-classical uncertainties, as we will see below. Thus, the quantum structural quantifier seems to “sense” that the correlation structure that it depicts is maximal as we enter the semi-classical domain. The left panel tells us that the structural quantifier becomes maximal when the vibrational energy equals the thermal–kinetic one.

Let us elaborate on this last result. Both the cases of T→∞ and T=0 (one has the vibrational energy ħ/2) have a zero structural quantifier *C*. The maximum *C* should be attained in a scenario that is “intermediate” between these two extreme instances. This happens precisely when the vibrational energy equals the thermal–kinetic one.

Figure 2 displays several thermal quantifiers versus uncertainty in two distinct fashions for didactic purposes. Notice that at the minimum minimorum (MM) uncertainty value, the entropy, specific heat, and structural quantifier all vanish.

It may be of some interest to see that all relevant thermal quantifiers can be cast in terms of $D=U_{r}=P\left(\hat{\rho }\right)$. Indeed, we have

(16) $$e^{-\beta \hbar \omega }=\frac{1-D}{1+D},$$

which implies that

(17) $$1-e^{-\beta \hbar \omega }=\frac{2D}{1+D}.$$

Therefore—and this is, we believe, a new way of casting HO thermal quantities—solely in terms of $D=U_{r}$, we have the panoply of expressions:

(18) $$S=\left(\frac{1-D}{2D}\right)\ln\left(\frac{1+D}{1-D}\right)+\ln\left(\frac{1+D}{2D}\right),$$

while the structural quantifier reads

(19) $$C=\left(\frac{1-D}{2}\right)\ln\left(\frac{1+D}{1-D}\right)+D\ln\left(\frac{1+D}{2D}\right),$$

the free energy turns out to be

(20) $$F=\hbar \omega \frac{\ln\left(\frac{\sqrt{1-D^{2}}}{2D}\right)}{\ln\left(\frac{1-D}{1+D}\right)},$$

the energy is

(21) $$U=\frac{\hbar \omega }{2D},$$

and the specific heat becomes

(22) $$C_{V}=\frac{1}{4}\left(\frac{1-D^{2}}{D^{2}}\right){\left[\ln\left(\frac{1+D}{1-D}\right)\right]}^{2}.$$

Finally, the number of particles becomes

(23) $$\langle \hat{n}\rangle =\frac{1-D}{2D}.$$

All of the HO thermodynamics can be expressed in terms of either *D* or the purity. These may be trivial, but they are novel results.

## 4. Extending Bridges to a Semi-Classical Environment

### 4.1. Introduction: Coherent States and Husimi Distributions

The well-known semi-classical Wehrl entropic quantifier *W* constitutes a phase space measure of localization [38,39]. It is constructed via coherent states |z〉 [38,40,41] and is regarded as a powerful tool in statistical physics. Remember that coherent states are eigenstates of an appropriate annihilation operator $\hat{a}$ that satisfy the relation $\hat{a}|z\rangle =z|z\rangle$ [41,42,43]. The definition of *W* is

(24) $$W=-\int \frac{dx\;dp}{2\pi \hbar }\mu \left(x,p\right)\;\ln\mu \left(x,p\right),$$

which is thus a Shannon-like information measure [44] to which Jaynes’ MaxEnt elaborations can be applied.

The Husimi distributions (HDs) μ(x,p) [45] are the diagonal elements of the density operator in the coherent-state basis |z〉. Accordingly,

(25) $$\mu \left(x,p\right)=\langle z|\hat{\rho }|z\rangle .$$

The μ are semi-classical distributions linked to a density matrix $\hat{\rho }$ for the system [41,42,43], normalized in the fashion

(26) $$\int \frac{dx\;dp}{2\pi \hbar }\;\mu \left(x,p\right)=1.$$

It is well known that μ(x,p) is a Wigner distribution $\rho_{W}$, smeared over an *ℏ*-sized region of phase space [40]. Such daubing makes μ(x,p)>0, although $\rho_{W}$ lacks such a positive character. The HD is a special sort of distribution, concerning an approximate specification of location in phase space [40]. The uncertainty principle acquires the form

(27) W≥1,

as conjectured by Wehrl [38] and proved by Lieb [46]. Equality is attained for $\hat{\rho }$ in a coherent state [38,46].

In considering *T* equilibrium states, one usually regards the system’s state as an incoherent mixture of eigenenergies $E_{n}$ weighted by the Boltzmann factor $\exp\left(-\beta E_{n}\right)$. Gibbs’s canonical distribution is the thermal density matrix given by $\hat{\rho }=\exp\left(-\beta E_{n}\right)/Z$ with $Z=\sum_{n}\exp\left(-\beta E_{n}\right)$.

If we want a *W* expression for the Hamiltonian $\hat{H}$ of eigenstates |n〉 and eigen-energies $E_{n}$, one can always write [40]:

(28) $$\mu \left(x,p\right)=\langle z|\hat{\rho }|z\rangle =\frac{1}{Z}\;\sum_{n}e^{-\beta E_{n}}\;{\left|\langle z|n\rangle \right|}^{2}.$$

A useful path *W* begins, then, with Equation (28) and follows with Equation (24). Distributions cast in terms of the coherent states |z〉 of the harmonic oscillator are useful in multiple contexts [40,41,42,43].

### 4.2. HO Specialization

The above ruminations are of a general nature. Let us specialize things for the HO, whose Hamiltonian reads

(29) $$\hat{H}=\hbar \omega \;\left({\hat{a}}^{†}\hat{a}+1/2\right)=\left(\hbar \omega /2\right)\left({\hat{a}}^{†}\hat{a}+\hat{a}{\hat{a}}^{†}\right).$$

The complex eigenvalues *z* of the destruction operator $\hat{a}$ are

(30) $$z=\frac{1}{2}\;\left(\frac{x}{\sigma_{x}}+i\frac{p}{\sigma_{p}}\right),$$

where *x* and *p* are scaled by their respective variances (σ) in the HO ground state $\sigma_{x}={\left(\hbar /2m\omega \right)}^{1/2}$, $\sigma_{p}={\left(\hbar m\omega /2\right)}^{1/2}$, and $\sigma_{x}\sigma_{p}=\hbar /2.$ Thus, the Husimi μ(x,p) becomes [40,47]

(31) $$\mu \left(x,p\right)\equiv \mu \left(z\right)=\left(1-e^{-\beta \hbar \omega }\right)\;e^{-\left(1-e^{-\beta \hbar \omega }\right){\left|z\right|}^{2}},$$

which is normalized according to Equation (26). In addition, the mean energy is [48]:

(32) $$\langle \hat{H}\rangle =\hbar \omega \left({\langle |z|}^{2}\rangle -\frac{1}{2}\right)=\hbar \omega \left(\frac{1}{1-e^{-\beta \hbar \omega }}-\frac{1}{2}\right)\equiv \frac{\hbar \omega }{2\tanh(\beta \hbar \omega /2)},$$

which coincides with its quantum counterpart.

The HO–Wehrl measure now acquires the appearance [47]:

(33) $$W=1-\ln\left(1-e^{-\beta \hbar \omega }\right).$$

## 5. HO–Semi-Classical Thermal Treatment and Uncertainty Relations

We enter our semi-classical contributions at this stage. The semi-classical disequilibrium is easily seen to be

(34) $$D_{sc}=D_{semi-quant}=\int \frac{d^{2}z}{\pi }\;\mu^{2}\left(z\right),$$

where $d^{2}z/\pi =d\left(Re\;z\right)d\left(Im\;z\right)=dxdp/\left(2\pi \hbar \right)$ is the differential *z* plane’s area element [41]. We will use the subscript sc for the semiclassical case.

After evaluation, that integral becomes

(35) $$D_{semi-quant}=\frac{1}{2}\left(1-e^{-\beta \hbar \omega }\right),$$

and by comparing to (17), we see that

(36) $$D_{semi-quant}=\frac{D}{1+D},$$

meaning that

(37) $$D_{semi-quant}\leq D,$$

an unsuspected relationship, which makes sense, however, since, as a classical density distribution (DD), one expects it to be closer to the uniform distribution than a quantal DD derived from a density operator.

The structural quantifier $C_{sc}=D_{sc}\;W$ derives from Equations (33) and (35) and reads

(38) $$C_{sc}=\frac{1}{2}\left(1-e^{-\beta \hbar \omega }\right)\left(1-\ln(1-e^{-\beta \hbar \omega })\right).$$

Thermal uncertainties express the effect of temperature on Heisenberg’s celebrated relations (see, for instance, [28,35,37,49]). We now use a result obtained in Ref. [40] (Equation (3.12)), where the authors cast Wehrl’s information measure in terms of the “coordinates” variances $\Delta_{\mu }x$ and $\Delta_{\mu }p$, obtaining

(39) $$W=\ln\left(\frac{e}{\hbar }\;\Delta_{\mu }x\;\Delta_{\mu }p\right).$$

In the present context, the relation $W=1-\ln(1-e^{-\beta \hbar \omega })$ allows us to write [47]:

(40) $$\Delta_{\mu }x\;\Delta_{\mu }p=\frac{\hbar }{1-e^{-\beta \hbar \omega }}.$$

In view of Equation (35), we can affirm that there is an exact semi-classical replica of the quantum equality (14) above, which reads

(41) $$D_{sc}\;\Delta_{\mu }x\;\Delta_{\mu }p=\frac{\hbar }{2}.$$

In addition, the structural quantifier has the form

(42) $$C_{sc}=\frac{\ln\left(\frac{e\;\Delta_{\mu }x\;\Delta_{\mu }p}{\hbar }\right)}{\Delta_{\mu }x\;\Delta_{\mu }p/\left(\hbar /2\right)}.$$

We note that when $\Delta_{\mu }x\;\Delta_{\mu }p=\hbar$, then $C_{sc}=1/2,$ which is the maximum possible value attained by $C_{sc}$. Figure 2 depicts the behavior of $C_{sc}$ in terms of the uncertainty relation $\Delta_{\mu }x\;\Delta_{\mu }p$. Note that *W* and its associated structural quantifier can be expressed exclusively in uncertainty terms.

**Figure 3:**
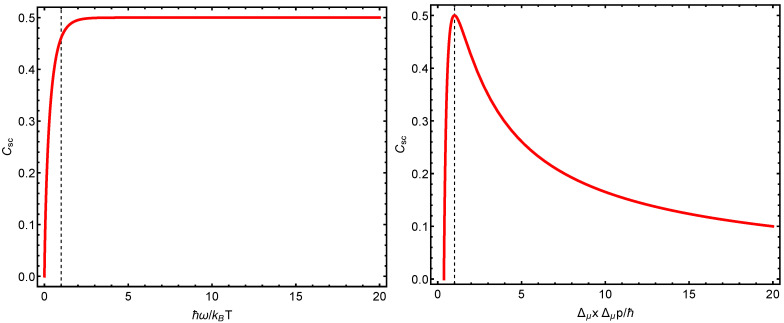
(**Left panel**): statistical complexity $C_{sc}$ versus $\hbar \omega /k_{B}T$. The vertical line indicates equality between the thermal and the vibrational energies. As the frequency ω grows relative to the temperature, the complexity becomes a constant. (**Right panel**): structural quantifier $C_{sc}$ versus $\Delta_{\mu }x\;\Delta_{\mu }p/\hbar$. The vertical line indicates optimal semi-classical localization.

In Figure 3, we notice that (i) the Wehrl structural quantifier attains its maximum values at the same place at which the quantal structural quantifier does. (ii) This place corresponds to the maximum possible semi-classical localization in phase space. (iii) Wehrl’s structural quantifier grows from zero vibrational energy (VE) till the VE becomes half the thermal-kinetic energy.

## 6. Possible Classical Extension

For completeness’ sake, we add here a word regarding the classical scenario. We obtain from Refs. [5,36,37] the three relations (note that we will use here the subscript class for the classical case):

(43) $$D_{class}=\beta \hbar \omega /2,$$

(44) $$S_{class}=1-\ln\left(\beta \hbar \omega \right)=1-\ln\left(2D_{class}\right),$$

vanishing thus for (here, *e* is the basis of natural logarithms)

(45) $$D_{class}=e/2,$$

and becoming negative is $D_{class}>e/2$, a typical classical artifact.

Finally, the special equality obeyed by the uncertainty relation can be extended to the classical realm. In this case, one has

(46) $$\Delta_{class}\;x\Delta_{class}p=\frac{\hbar }{\beta \hbar \omega }=\frac{\hbar }{2D_{class}},$$

or, significantly enough, we have a classical counterpart of the quantum equality (14) that reads

(47) $$D_{class}\;\Delta_{class}\;x\Delta_{class}p=\frac{\hbar }{2}.$$

A word of caution may be pertinent here. In this instance, *ℏ* is just an arbitrary elementary action that one introduces in classical statistical mechanics in order to avoid Gibbs’ paradox. It is gratifying, though, that (47) preserves the structure of Equation (14).

Using relation (16), we see that

(48) $$D_{class}=\ln{\left(1-D\right)}^{1/2}-\ln{\left(1+D\right)}^{1/2},$$

so that $D_{class}$ vanishes if its quantum counterpart does so. However, it diverges when *D* attains its maximum value of unity. In addition, with some algebra, the classical structural quantifier can be written in terms of the quantum uncertainties as

(49) $$C_{class}=\frac{\ln\left(e\;\Delta_{class}x\Delta_{class}p/\hbar \right)}{2\Delta_{class}x\Delta_{class}p/\hbar }.$$

**Figure 4:**
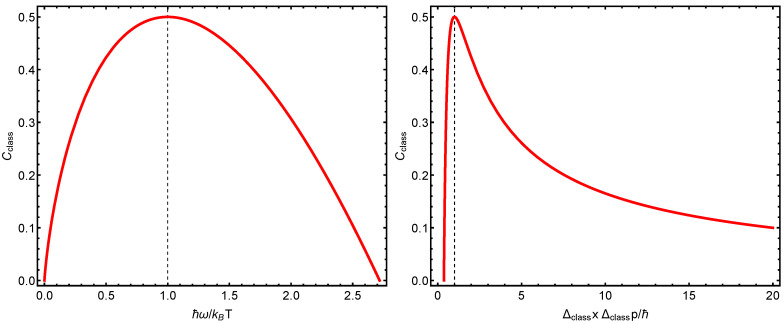
(**Left panel**): structural quantifier $C_{class}$ versus $\hbar \omega /k_{B}T$. The maximum is attained at $\hbar \omega /k_{B}T=1$, that is, equality between thermal and vibrational energies. (**Right panel**): structural quantifier $C_{class}$ versus $\Delta_{class}x\Delta_{class}p=\hbar$. Remarkably enough, $C_{class}$ is maximal at the same uncertainty values that maximize its quantum counterpart.

**Figure 5:**
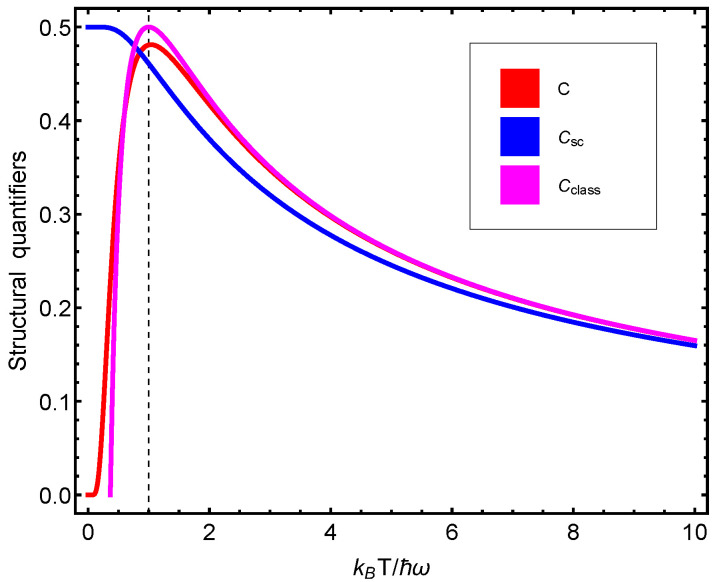
Our three manners of calculating complexities (or structural quantifiers), *C* versus $k_{B}T/\hbar \omega$. The vertical line signals equality between the thermal and vibrational energies. As the temperature grows, the three manners tend to yield identical results.

We illustrate things in Figure 4 and Figure 5. Notice that, also classically, the structural quantifier becomes maximal when the two types of energies at play become equal. Note the extraordinary similitude between the quantum and the classical *C* structural quantifiers.

## 7. Application to a Nuclear Physics Model

We apply here our thermal quantifiers *S*, *D*, and *C* to a fermion model system used in nuclear physics [16,17,18,19,20].

### 7.1. The Model

The Lipkin model (LM) [22] was very useful in research that revolved around the validity and/or usefulness of several theoretical techniques devised for investigating multiple facets of the fermion many-body problem. The LM is based on an SU(2) algebra. The properties of the pertinent solutions can be investigated via group-theory techniques. We will occupy ourselves here with an LM version proposed in Ref. [16].

The *N* fermion models of [16,22] treat *N* fermions distributed between (2N)-fold degenerate single-particle levels, whose energy separation is a gap ϵ. Two quantum numbers (qn) (mu and *p*) are allocated to a generic single-particle (sp) state. The μ qn adopts the values μ=−1 (lower level) and μ=+1 (upper level). The *p* qn, often referred to as a quasi-spin or pseudo-spin, picks out a specific *p* value from the *N*-fold degeneracy. The pair p,μ may be viewed as a ”site” that is either occupied or empty. One has

(50) N=2J,

with *J* standing for an “angular momentum”.

### 7.2. Second Quantization Language

We need the creation and destruction operators $C^{†}$ and *C*. Following Lipkin et al. [22], we introduce the quasi-spin operators

(51) $${\hat{J}}_{+}=\sum_{p}C_{p,+}^{†}C_{p,-},$$

(52) $${\hat{J}}_{-}=\sum_{p}C_{p,-}^{†}C_{p,+},$$

(53) $${\hat{J}}_{z}=\sum_{p,\mu }\mu \;C_{p,\mu }^{†}C_{p,\mu },$$

(54) $${\hat{J}}^{2}={\hat{J}}_{z}^{2}+\frac{1}{2}\;\left({\hat{J}}_{+}{\hat{J}}_{-}+{\hat{J}}_{-}{\hat{J}}_{+}\right),$$

where the eigenvalues of ${\hat{J}}^{2}$ equal J(J+1).

### 7.3. Hamiltonian *H* for Our Model

This has a coupling constant $V_{s}$ and reads [16]:

(55) $$\hat{H}=\epsilon {\hat{J}}_{z}-V_{s}\left(\frac{1}{2}\left({\hat{J}}_{+}{\hat{J}}_{-}+{\hat{J}}_{-}{\hat{J}}_{+}\right)-\hat{J}\right),$$

or, with $V=V_{s}/\epsilon$ (equivalently, ϵ=1). Moreover,

(56) $$\hat{H}={\hat{J}}_{z}-V\left(\frac{1}{2}\left({\hat{J}}_{+}{\hat{J}}_{-}+{\hat{J}}_{-}{\hat{J}}_{+}\right)-\hat{J}\right),$$

and the unperturbed ground state (gs) for V=0 is, given Equation (50),

(57) $$|J,J_{z}\rangle =|J,-N/2\rangle ,$$

whose energy $E_{o}$ is

(58) $$E_{o}=-N/2.$$

Doubly occupied *p* sites are not permitted. $\hat{H}$ commutes with the two operators ${\hat{J}}^{2}$ and ${\hat{J}}_{z}$.

Thus, the exact solution must be located within the *J*-multiplet of the unperturbed ground state. The states of this multiplet are called |J,M〉. One of them should then minimize the energy. The concomitant *M* value depends on the value of *V*.

### 7.4. Phase Transitions

A remarkable feature of the model is that, as *V* grows from zero, $E_{o}$ is not immediately modified. It keeps its value until a critical *V*-specific value is reached, which equals 1/(N−1). At this stage, the interacting ground state suddenly becomes |J,−N/2+1〉. If *V* keeps growing, additional phase transitions (pt) occur. Between $J_{z}=-k$ and $J_{z}=-k+1$, this takes place at V=1/(2k−1). The pt series ends as the interacting ground state becomes either $J_{z}=0$ ($V_{crit}=1$ for integer *J*) or $J_{z}=-1/2$ ($V_{crit}=1/2$ for odd *J*). In such cases, we have, independently of the value *J* [16],

(59) $$V_{crit}=1/2,$$

for half *J* and

(60) $$V_{crit}=1,$$

for integer *J*.

### 7.5. Finite Temperature

One needs to study model results for different *J* values, which is easy because double occupancy of a *p* site is strictly forbidden. Thus, the Hamiltonian matrix should be the (2J+1)×(2J+1) one of the $J_{z}=-N/2$ multiplet, with N=2J [22].

One has for the free energy F(J) in terms of the partition function Z(J):

(61) $$F=-T\ln Z=-T\ln\mathrm{Tr}(\exp\left(-\beta \hat{H}\right)),$$

where we set the Boltzmann constant equal to unity. For each different *J*, the trace operation is a sum over the $J_{z}$ quantum number *m* and

(62) $$Z\left(J\right)=\sum_{m=-J}^{m=J}\;\;\exp\left(-\beta E_{m}^{J}\right),$$

with an energy $E_{m}^{J}$ [16]

(63) $$E_{m}^{J}=m-V\left[J\left(J+1\right)-m^{2}-J\right].$$

The associated Gibbs canonical ensemble probabilities $P_{m}^{J}$ are then [50]:

(64) $$P_{m}^{J}=\frac{\exp\left(-\beta E_{m}^{J}\right)}{Z(J)},$$

for all m=−J,−J+1,…,J−1,J, and the Boltzmann–Gibbs *S* entropy becomes [50]:

(65) $$S\left(J\right)=-\sum_{m=-J}^{m=J}\;\;P_{m}^{J}\ln P_{m}^{J}.$$

As for the number of micro-states *m* one has, of course,

(66) O(J)=2J+1,

so that the uniform probabilities become

(67) $$P\left(u_{J}\right)=1/O\left(J\right).$$

Our disequilibrium is then

(68) $$D\left(J\right)=\sum_{m=-J}^{m=J}{\left[P_{m}^{J}-P\left(u_{J}\right)\right]}^{2}.$$

Consequently, the pertinent statistical complexity *C* becomes

(69) C(J)=D(J)S(J).

One expects that *C* will display a maximum at the phase transitions [5].

### 7.6. Application Results

**Figure 6:**
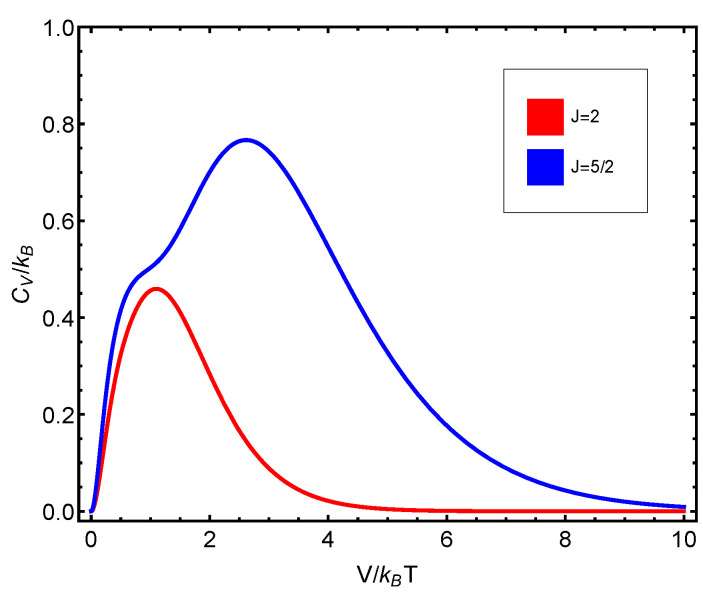
$C_{V}$ versus V/T for N=45.

**Figure 7:**
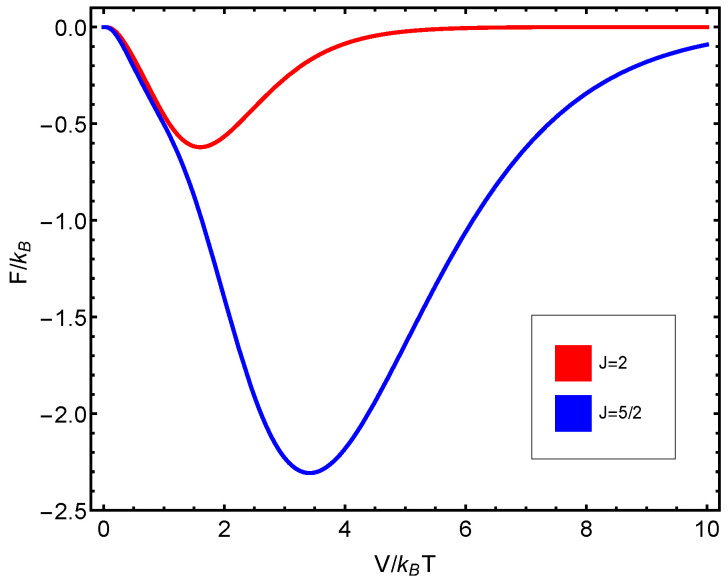
F) versus V/T for N=45.

**Figure 8:**
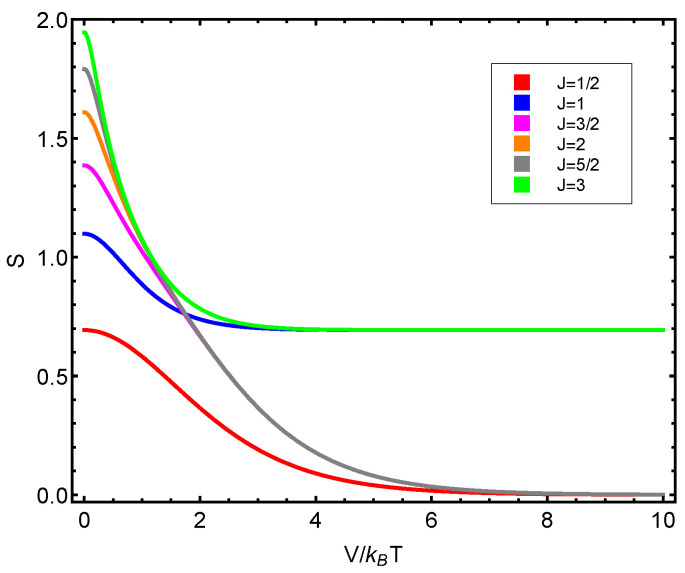
S(J) versus V/T for different *J* values. Since the fermion number N=2J, we detect a significantly distinct behavior according to whether the fermion number is even or odd.

**Figure 9:**
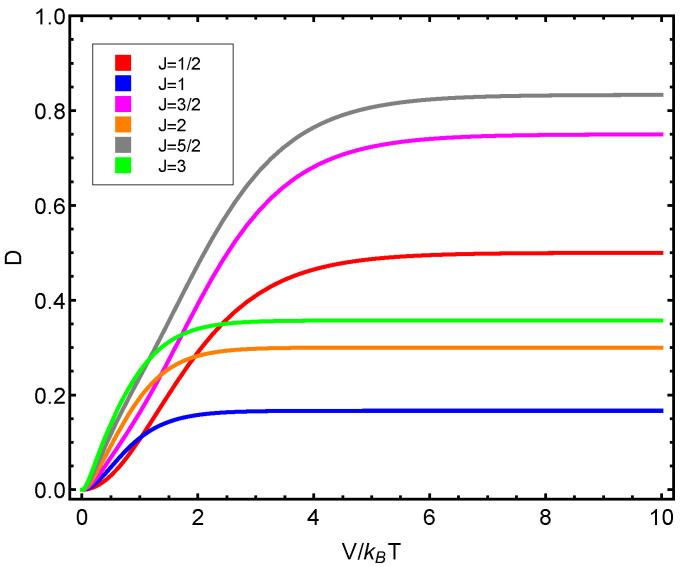
D(J) versus V/T for different *J* values. Since the fermion number N=2J, we detect a significantly distinct behavior according to whether the fermion number is even or odd.

**Figure 10:**
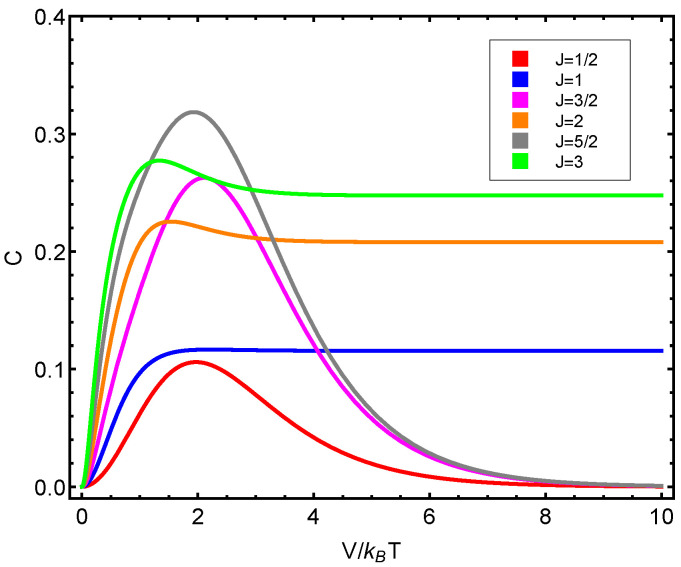
C(J) versus V/T for different *J* values. This displays a maximum that signals the phase transition. Since the fermion number N=2J, we detect a significantly distinct behavior according to whether the fermion number is even or odd.

**Figure A1:**
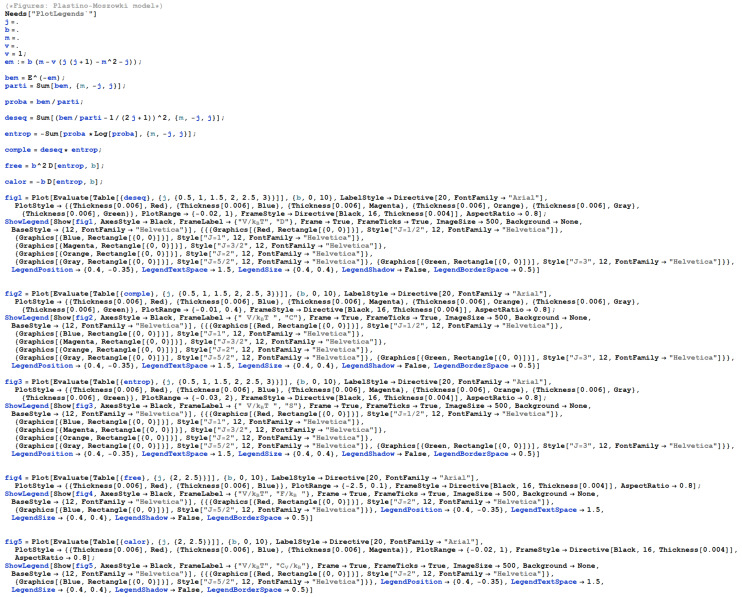
Our computer program

The numerical progtam utilized can be inspected in Figure A1 in Appendix A. Also, see Figure 6, Figure 7, Figure 8, Figure 9 and Figure 10. Just to show how the model works, we depict the free energy *F* (Figure 7) and the specific heat $C_{V}$ (Figure 6) versus V/T.

Afterwards, we plot the three quantifiers S(J), D(J), and C(J) below as a function of the ratio between the coupling constant *V* and the temperature *T*. We see that the three quantities behave in a quite different manner according to whether the number of fermions in the system is even or odd. These odd–even effects have their counterparts in nature [51,52,53].

As stated above, our odd–even effects detected here have their counterparts in nature [51,52,53]. The odd–even staggering of nuclear binding energies is well known [53]. A rather similar effect can be found in other finite fermion systems. The staggering in nuclei and grains is attributed mainly to pairing correlations. In clusters, it originates from the Jahn–Teller effect (see [51,52,53] and the references therein). These odd–even differences in nuclear masses are also influenced by mean-field and odd-nucleon blocking effects [54].

## 8. Conclusions

This work had several parts. In the first part, we uncovered and/or found some novel facets of information-related Gibbs statistical descriptions. More specifically, for mixed states (at a temperature *T*), we established the equality

ΔxΔpP=ΔxΔpD=ħ/2.

This equality was suitably extended, mutatis mutandis, to both the semi-classical and the classical realms, as we explained above. Further,

- At the minimum minimorum uncertainty value, the entropy, specific heat, and structural quantifier *C* all vanish.
- There is a strong connection between the disequilibrium *D* and the thermal uncertainty (TU). As *D* grows, the TU decreases. The TU is minimal for pure states where D=1.
- Note that all quantities involved in (15) are observable (in principle), so we are dealing with a relation that has its counterpart in nature.

The second part of this effort involved semi-classical and classical scenarios. We highlight that

1. The Wehrl structural quantifier $C_{sc}$ attains its maximum values at the same place at which the quantal structural quantifier *C* does so.
2. This place corresponds to the maximum possible semi-classical localization in phase space.
3. Wehrl’s structural quantifier $C_{sc}$ grows from zero at null vibrational energy (VE) until the VE attains half of the thermal–kinetic energy, and then remains constant.
4. $\Delta_{\mu }x\;\Delta_{\mu }p/\hbar$ can be regarded as the phase-space localization error *e* (in its natural units) that accompanies the Husimi distribution.
5. The Wehrl structural quantifier $C_{sc}$ becomes a maximum in these circumstances.
6. We emphasize that $C_{V}$ attains its constant classical value as soon as the thermal energy equals the vibrational one.
7. The three different structural quantifiers, *C*, at play in this work behave in a remarkably similar fashion, as shown in the last graphs.

The third part referred to the application of the three different structural quantifiers *S*, *D*, and *C* to a nuclear physics model, where the quantifiers permit one to find significant fermionic differences. 

## Abbreviations

The following abbreviations are used in this manuscript:

LMC	Lopez-Rioz, Mancini, and Calbet
LMCTSQ	Lopez-Rioz, Mancini, and Calbet thermal structural quantifiers
TUR	Thermal uncertainty relation
HO	Harmonic oscillator
MM	Minimum minimorum
TQF	Thermal quantum quantifiers
HD	Husimi distributions
DD	Density distribution
LM	Lipkin model

## Appendix A. Mathematics Program

It is given in Figure A1 below.
